# Corrosion of copper nickel titanium archwire in chlorhexidine,  sodium fluoride, and chitosan mouthwashes

**DOI:** 10.12688/f1000research.129043.3

**Published:** 2024-05-30

**Authors:** Erliera Sufarnap, Kholidina Imanda Harahap, Ika Devi Adiana, Davin Lim, Chatty Lim, Christy Christy

**Affiliations:** 1 Orthodontic Department, Faculty of Dentistry, Universitas Sumatera Utara, Medan, North Sumatera, 20155, Indonesia; 2 Department of Dental Material, Faculty of Dentistry, Universitas Sumatera Utara, Medan, North Sumatera Utara, 20155, Indonesia; 3 Department of Pediatric Dentistry, Univsersitas Sumatera Utara, Medan, North Sumatera Utara, 20155, Indonesia; 4 Residence of Orthodontic, Faculty of Dentistry, Universitas Sumatera Utara, Medan, North Sumatera, 20155, Indonesia; 5 Profession Student, Faculty of Dentistry, Universitas Sumatera Utara, Medan, North Sumatera, 20155, Indonesia

**Keywords:** CuNiTi archwire, nickel ion release, copper ion release, surface topography, deflection, unloading force, chlorhexidine, and natrium fluoride.

## Abstract

**Background:**

Copper (Cu), nickel (Ni), chromium (Cr) ion release, and surface topography change from the orthodontic wire are the initial processes of corrosion that may affect the mechanical properties of the archwire. In this study, we aim to evaluate the effect of CHX, NaF, and chitosan on the corrosion of CuNiTi wire nickel and copper ions released, surface roughness change, and archwire deflection.

**Methods:**

Ninety samples of CuNiTi Tanzo™ archwires were divided into five groups according to their immersion solution: Artificial Saliva, CHX, NaF, CHX-NaF, and chitosan group. Each group was further divided into three subgroups (n=6) corresponding immersion time, *i.e.,* two, four, and six weeks. The corrosion of the samples was analyzed with an atomic absorption spectrophotometer (AAS), scanning electron microscope (SEM), and universal testing machine (UTM).

**Results:**

The amount of nickel ion releases was increasing, but the copper ion releases were reduced by the time of observations. The highest nickel ion was released in the CHX-NaF group and the lowest in the chitosan group for six-week immersion. It also corresponded to the surface topography by SEM analysis which showed the most extended cracks and deep pits in the CHX-NaF group and a smoother surface in the chitosan group. Copper ion release showed the highest ion release in the NaF group and the lowest release in the chitosan group. The unloading force of CuNiTi archwire deflection remains the same at week two and week four for all mouthwashes.

**Conclusion:**

The use of mouthwashes that contained CHX, NaF, and chitosan could further alter the passive layer and cause higher nickel and copper ion release and increased CuNiTi archwire surface structure porosity. But there is no distinction between mouthwashes to release the unloading force within two until four weeks.

## Introduction

Nickel-titanium alloy archwire offers greater flexibility and resistance to deformation and also exhibits excellent biocompatibility and corrosion resistance ( Mitchell, 2013). Further addition of copper (Cu) in Cu nickel (Ni) titanium (Ti) archwires produces a more constant force on the teeth ( Singh, 2007; Bhalajhi, 2012; Phulari, 2017). However, in the oral environment, archwires are constantly exposed to various stresses from masticatory forces, loading appliances, temperature fluctuations, and varieties of ingested food and saliva ( Chaturvedi and Upadhayay, 2010).

The electrochemical mechanism of corrosion plays an essential role in metal corrosion when in contact with an electrolyte fluid, for example, saliva or mouthwashes. When two different alloys are in contact with a fluid electrolyte, the alloy with lower electrode potential will become the anode and produce oxidation that released several ions into the solution ( Anusavice *et al.*, 2012). Corrosion creates two issues; it can alter the physical properties of archwires ( Mane *et al.*, 2012; Geramy, Hooshmand and Etezadi, 2017; Doddamani, Ghosh and Tan, 2018) and cause a local or systemic condition due to allergic reactions and biological side effects ( Lü *et al.*, 2009; Pazzini *et al.*, 2009; Suárez *et al.*, 2010; Hafez *et al.*, 2011; Castro *et al.*, 2015). The metal products released during corrosion are nickel, chromium, and copper. Nickel is classified as a chemical carcinogen ( IARC Working Group, 1990). In addition, it is a powerful medium for an immune reaction which can lead to a hypersensitivity reaction, contact dermatitis, gingivitis, gingival hyperplasia, periodontal stomatitis, periodontitis, burning mouth syndrome, angular cheilitis, cytotoxicity, mutagenic reaction ( Hafez *et al.*, 2011; Heravi *et al.*, 2015; Lü *et al.*, 2009). Meanwhile, copper is required by the body, but an excessive amount can produce cytotoxicity in the form of allergies at the point of contact and metal deposits in organs ( Farrukh, 2011; Mahalaxmi, 2013).

Deflection of the archwire also plays an essential role in affecting tooth movement during orthodontic treatment ( Aghili *et al.*, 2017; Khatri and Mehta, 2014). Deflection of the archwire is defined by the ability of the archwire to transmit forces to the dentoalveolar to promote tooth movement ( Parvizi and Rock, 2003; Sarul *et al.*, 2013).

Dental hygienist usually prescribes mouthwashes to patients with low oral hygiene and a high risk of caries to prevent the formation of microbial plaque ( Anuwongnukroh *et al.*, 2017; Castro *et al.*, 2015). The use of fluoride mouthwashes helps the enamel layer remineralize and protects it from the acidic environment ( Roveri *et al.*, 2009; Sivapriya *et al.*, 2017), but the production of hydrofluoric acid (HF) may have a destructive impact on the archwires. HF degrades the protective oxide layers on the surface, which leads to corrosion ( Castro *et al.*, 2015; Hafez *et al.*, 2011; Lü *et al.*, 2009; Marques *et al.*, 2012; Suárez *et al.*, 2010). But there are several published studies on the corrosion resistance of NiTi alloys in saliva and NaF solutions even with increasing concentrations of fluorides ( Heravi, Hadi Moayed and Mokhber, 2015; Mirhashemi, Jahangiri and Kharrazifard, 2018; Fatene *et al.*, 2019).

Chlorhexidine also has high effectiveness in preventing the formation of dental plaque and is also effective in decreasing gingival inflammation ( Metin-Gürsoy and Uzuner, 2014; Deriaty, Nasution and Yusuf, 2018). Several authors have evaluated a significant lowering in corrosion resistance in stainless steel or NiTi archwires in chlorhexidine mouthwashes compared to other mouthwashes ( Danaei *et al.*, 2011; Deriaty *et al.*, 2018; Habar and Tatengkeng, 2020). Several studies also showed degradation in the performance of an elastomeric chain ( Sufarnap *et al.*, 2021), and more significant surface corrosion was observed under the scanning electron microscope (SEM) in wires from chlorhexidine mouthwashes ( Mane *et al.*, 2012; Doddamani, Ghosh and Tan, 2018; Chitra, Prashantha and Rao, 2020).

Chitosan is a natural polysaccharide resulting from the deacetylation of chitin. Chitosan has a broad antibacterial content and a low level of toxicity, so it is often used as a mouthwash for plaque control ( Chen and Chung, n.d.; Fei Liu *et al.*, 2000). A study by Uraz *et al.* showed no significant difference in the use of chitosan mouthwash compared to chlorhexidine mouthwash plus chitosan in decreasing plaque index ( Uraz *et al.*, 2012).

Several studies were conducted on the corrosion resistance, deflection, and ion release of NiTi orthodontic archwires in chlorhexidine or fluoride mouthwashes. However, there were limited studies observing nickel ion release, copper ion release, deflection test, and surface structure in mouthwashes containing chlorhexidine and fluoride, and chitosan in CuNiTi archwire, and as such this became the objective of this study. We hypothesized the differences found at each immersion solution at each time observation to the surface structure, deflection, and nickel and copper ion release. This study is a continuation of the Devi *et al.* (2022) study.

## Methods

### Samples

The research type was an experimental study using a post-test control design. There were ninety (90) Tanzo (American Orthodontics ^®^) CuNiTi 4cm long archwires, sized 0.016×0.022 inches. Samples were divided into five groups according to the immersion solution, *i.e.,* control group, CHX group, NaF group, CHX-NaF group, and chitosan group. The samples were further divided into three subgroups (n=6) corresponding to the duration of immersion, two, four, and six weeks. The sample size was determined with the formulation from Irmawantini, 2017:

(n – 1).(r – 1) ≥ 15

(n – 1).(5 – 1) ≥ 15

4 *n* ≥ 19 *n* = 4.75 ( *minimum*)

### Mouthwash immersion phase

Each group has 18 samples divided into three subgroups (n=6) according to the immersion time: two, four, and six weeks. Group 1: immersed in artificial saliva as a control group (produced by the Oral Dental Hospital of Universitas Sumatera Utara Pharmacies followed by McDougall’s recipe of minerals: NaHCO _3_, Na _2_HPO _4_.12H _2_O, NaCl, KCl, CaCl _2_ anhydrous, MgCl _2_ anhydrous, and H _2_O) ( Khan *et al.*, 2021); Group 2: immersed in artificial saliva and 0.1% chlorhexidine gluconate mouthwash (Minosep, Minorock, Indonesia; as the CHX group); Group 3: immersed into artificial saliva and 0.05% NaF (Merck KGaA, Darmstadt, Germany, as the NaF group); Group 4: immersed into artificial saliva, 0.05% NaF, and 0.12% chlorhexidine gluconate (PerioKin ^®^; as the CHX-NaF group); Group 5: immersed into artificial saliva and 2% chitosan (prawn shells was formulated at the Laboratory of Research Centre (Faculty of Mathematics and Science, Universitas Sumatera Utara as the chitosan group). Ninety samples were made in total, and they were all incubated at 37°C.

Archwires were simulated in the mouth environment, all samples were immersed in 10 ml saliva within observation time (2,4, and 6 weeks), and mouthwashes were simulated two times a day for one minute. All samples immersed corresponding to the subgroup 2, 4, and 6 weeks; Minosep ^®^ mouthwash, 0.05% NaF, PerioKin ^®^, and chitosan mouthwash were added into the test tubes in group 2 to 5 simulated respectively for 28 minutes at two weeks subgroup, 56 minutes at four weeks subgroup, and 84 minutes at six weeks subgroup. The samples were agitated with a vortex for one minute before being incubated. After being immersed at each time point, wires were removed from the solutions, washed with distilled water, and dried for further surface roughness topography analysis with the SEM machine. Test tubes were sealed again with aluminum foil and placed at room temperature to prepare the analysis.

### Experiment analysis and measurement phase

The research was conducted at the Faculty of Pharmacies Laboratory, Universitas Sumatera Utara, where the samples were incubated at 37°C; nickel and copper ion release sample’s immersed solution were analyzed at Balai Standardisasi dan Pelayanan Jasa Industri (Baristand) Medan using atomic absorption spectrometry (AAS, Shimadzu AA7000); the surface structure of CuNiTi wires was tested with a scanning electron microscope (SEM, Hitachi TM3000, Tabletop Microscope, Japan) at 2000× magnification on three sites at Integrated Research Laboratory- Universitas Sumatera Utara; and deflection test with the Universal Testing Machine (UTM, Tensilon RTF 1350) at the Impact Fracture Research Center (IFRC) Laboratory, Faculty of Engineering, Universitas Sumatera Utara.

The SEM images were taken 3 times for each sample. The Region of interest (ROI) of the images captured within the roughest area. The AAS analysis had been taken one time with the Relative Percent Difference (%RPD) below 5%. While the UTM machine results also came with one-time measurements from the machine. Calibrators had been taken 5 times to get the optimal normal curve. The sample which had improper results or outranged the standard or normal curved had been recalculated. The results needed to analyse with the inter- or intra-reliability tests.

### Statistic analysis

Statistical analysis was performed using Statistical Package for Social Science (SPSS) 26.0 edition with Shapiro-Wilk for normality test (p≤0.05). The data obtained were analyzed statistically using the Kruskal-Wallis test to compare the amount of unloading force, nickel, and copper release in weeks two, four, and six.

## Results

**Figure 1.**
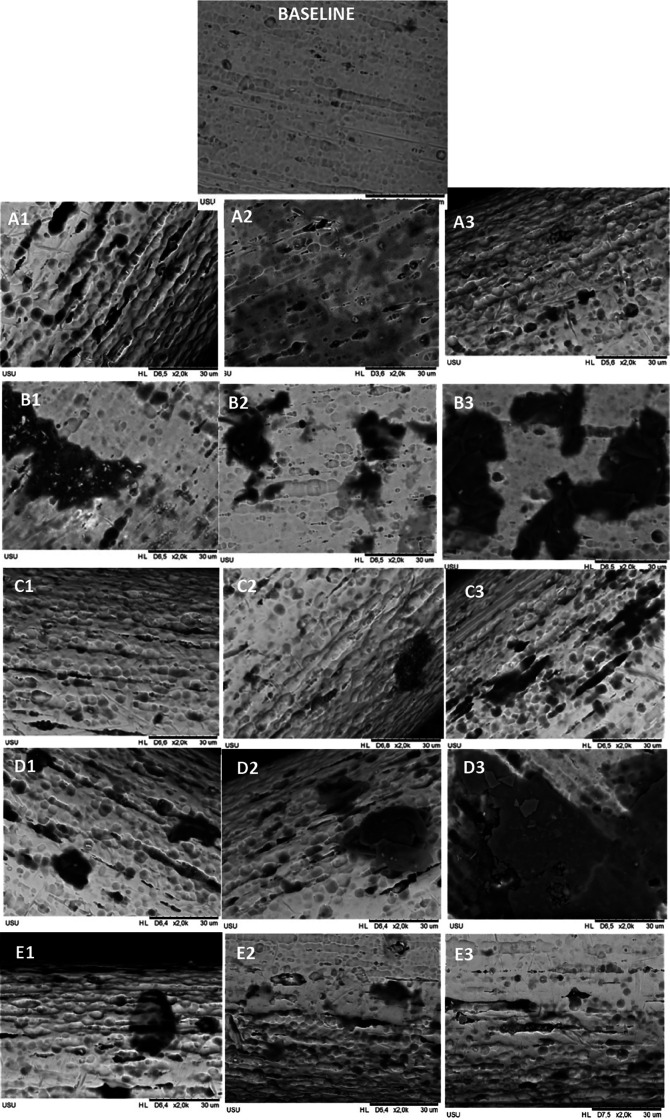
Surface roughness of CuNiTi archwire with SEM in 2000 magnification. Immersed with A. Control Group B. CHX Group C. NaF Group D. CHX+NaF Group E. Chitosan Group. (1,2,3 represent 2, 4, and 6 weeks).

Mean levels of nickel and copper released in each group for every time observation were significantly different, and the data are shown in Tables 1 and 2, respectively ( Sufarnap, 2022). Scanning electron microscope (SEM) images result after six weeks of immersion (the longest time) of CuNiTi’s wire surface topography are shown in Figure 1. The roughness was found in all groups, but the most extended surface defects, such as cracks and pits, were found in the CHX-NaF group, followed by more comprehensive pits in the CHX group compared to another group.

**Table 1.** Release of Nickel ion from Copper Nickel Titanium wires at different time intervals.

Solution	Mean (ppm in microgram/liter)±SD		
	Week two	Week four	Week six
Control Group	4.85±0.29	9.85±0.19	12.85±0.13
CHX Group	6.01±0.14	10.16±0.08	13.71±0.09
NaF Group	5.78±0.03	10.03±0.01	13.28±0.01
CHX-NaF Group	7.98±0.38xx	11.13±0.12	20.20±0.38
Chitosan Group	8.48±0.37	9.29±0.40	11.15±0.94
***p-value***	**0.001** ^**a**^	**0.001** ^**a**^	**0.001** ^**a**^

Data from five groups are expressed as mean±standard deviation (SD) (n=10). Superscript letters are significantly different by the *Kruskal Wallis* test (p<0.05). NaF: Sodium Fluoride, CHX: Chlorhexidine, CHX-NaF: Chlorhexidine-Sodium Fluoride.

**Table 2.** Release of Copper ion from Copper Nickel Titanium wires at different time intervals.

Solution	Mean (ppm in microgram/liter)±SD		
	Week-two	Week-four	Week-six
Control Group	1.62±0.03	1.41±0.04	1.28±0.03
CHX Group	2.38±0.04	2.24±0.08	2.20±0.05
NaF Group	3.89±0.001	3.54±0.001	3.23±0.001
CHX-NaF Group	3.37±0.07	3.06±0.10	3.00±0.06
Chitosan Group	1.07±0.04	1.07±0.04	0.97±0.02
*p-value*	0.001 ^a^	0.001 ^a^	0.001 ^a^

Data from five groups are expressed as mean±standard deviation (SD) (n=6). Superscript letters are significantly different by the *Kruskal Wallis* test (p<0.05). CHX: Chlorhexidine, CHX-NaF: Chlorhexidine-Sodium Fluoride, Chitosan: Chitosan 2%.

**Figure 2.**
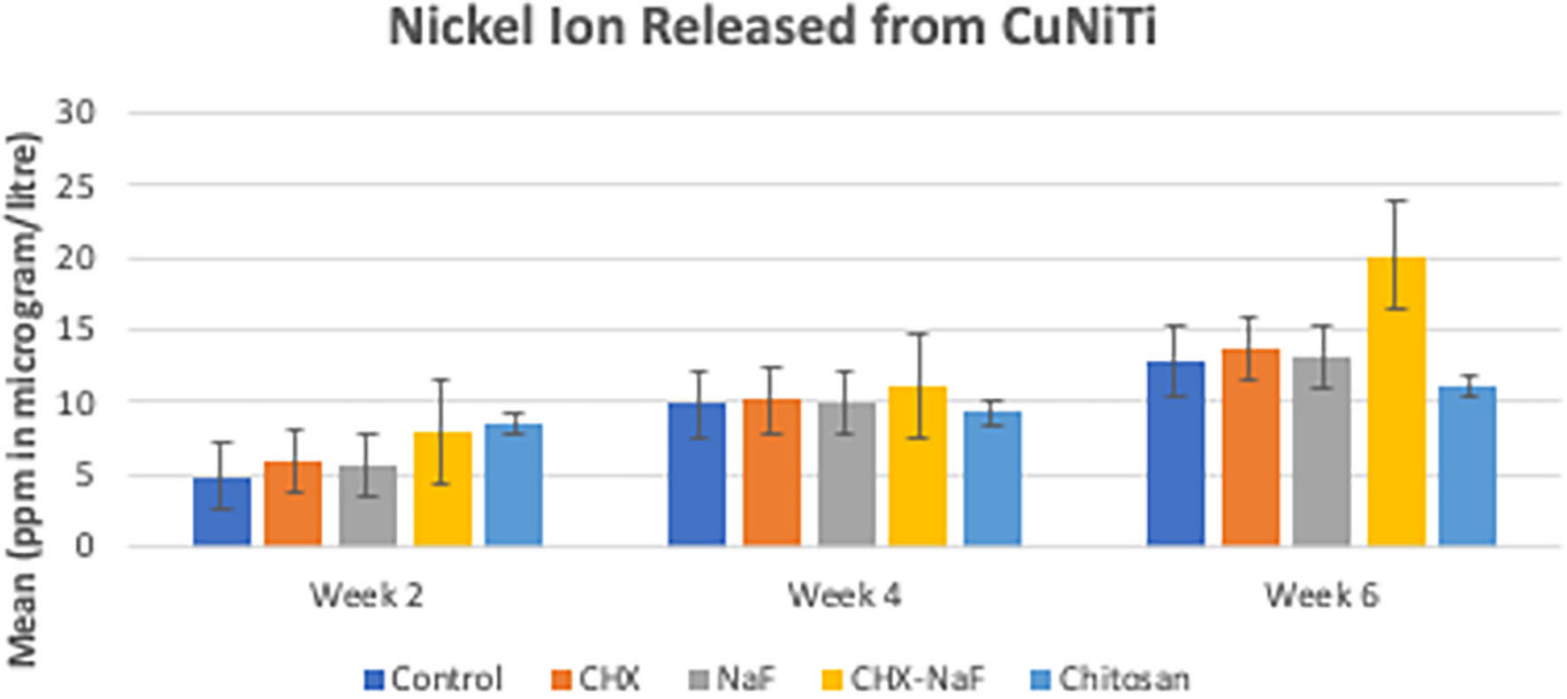
Comparison of Nickel released (ppm in μg/L) from CuNiTi wires at different time intervals. Abbreviations: NaF: Sodium Fluoride; CHX: Chlorhexidine; CHX-NaF: Chlorhexidine-Sodium Fluoride.

According to the result, nickel ions are increasingly released by the time of observation in all groups. In the beginning, the highest nickel released was at the chitosan group, but it had a slow release. The highest amount of nickel release also corresponded to the surface structure, which was the most prolonged observation in group 4 (CHX-NaF) ( Figure 2) ( Sufarnap *et al.*, 2022).

**Figure 3.**
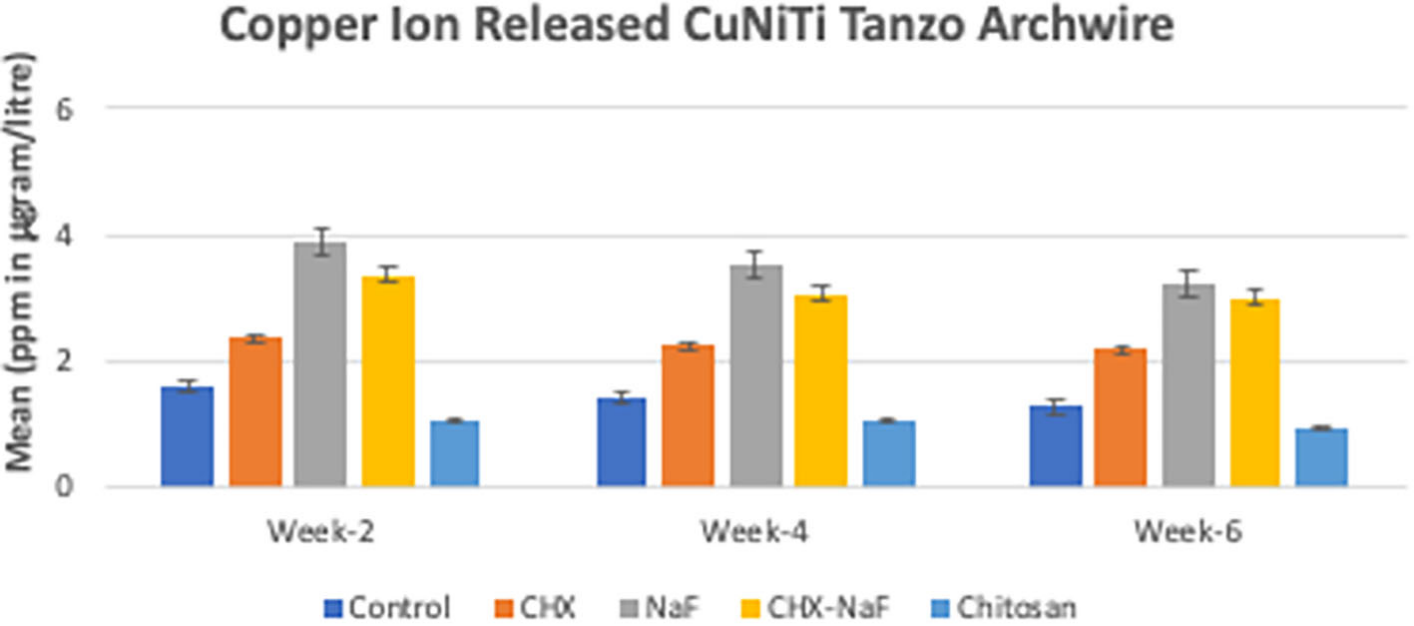
Comparison of Copper released (ppm in μg/L) from CuNiTi wires at different time intervals. Abbreviations: NaF: Sodium Fluoride; CHX: Chlorhexidine; CHX-NaF: Chlorhexidine-Sodium Fluoride.

**Figure 4.**
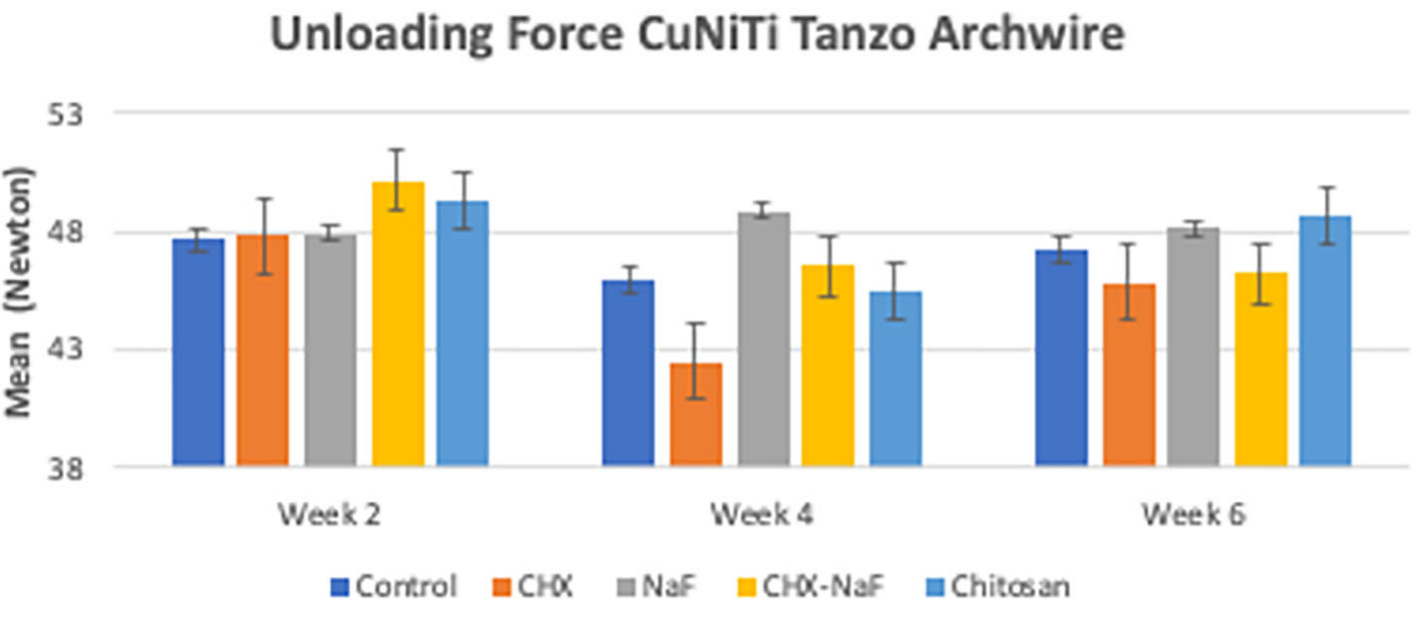
Comparison of unloading deflection force from CuNiTi wires at different time intervals. Abbreviations: NaF: Sodium Fluoride; CHX: Chlorhexidine; CHX-NaF: Chlorhexidine-Sodium Fluoride.

Copper ions released showed reduced by the time of observation at all groups. The highest copper released was found in the NaF group for all observation times ( Figure 3) ( Sufarnap *et al.*, 2022). The last analysis was the mean levels of unloading forces in each group. They are shown in Table 3. Based on the results, there were no significant differences in unloading forces at two weeks and four weeks of all groups but showed a significantly different in six-week group. The data are shown in Table 3 and Figure 4 ( Sufarnap *et al.*, 2022).

**Table 3.** Unloading forces from Copper Nickel Titanium wires at different time intervals (the baseline value was 45.049 Newton (N)).

Solution	Mean (N)±SD			
	Week two	Week four	Week six	*p-value* **
Control Group	47.639±2.1234	45.936±1.7143	47.207±2.2875	**0.262**
CHX Group	47.798±3.7015	42.532±8.8856	45.864±2.0239	**0.532**
NaF Group	47.95±2.32	48.89±1.23	48.14±1.34	**0.623**
CHX-NaF Group	50.141±1.3432	46.546±2.7308	46.224±1.1263	**0.010**
Chitosan Group	49.349±0.7325	45.428±1.7099	48.621±0.8625	**0.003**
***p-value*** *	**0.195**	**0.086**	**0.023**	

Data from five groups are expressed as mean±standard deviation (SD) (n=6).

* *p-value* between mouthwashes;

** *p-value* between Kruskal Wallis test (p<0.05). NaF: Sodium Fluoride, CHX: Chlorhexidine, CHX-NaF: Chlorhexidine-Sodium Fluoride.

## Discussion

The highest amount of nickel ion was 0.2020 mg from the CHX-NaF group at the six-week group, and the highest amount of copper ion was 0.03377 mg from the CHX-NaF group at week two. This showed the highest concentration of both ions was still below the warned concentration limit. The average amount of nickel intake obtained from food is 300 μg–500 μg/day ( Alarifi *et al.*, 2013; Milheiro *et al.*, 2016). While a nickel concentration of 600 μg–2500 μg can induce an allergic reaction, and a copper concentration of 10 ppm can cause a cytotoxic reaction ( Danaei *et al.*, 2011; Milheiro *et al.*, 2016). The, nickel and copper ions in this study were still released in the artificial saliva (control group), although it had the least amount compared to the other group. The mechanical properties of CuNiTi archwire consists 46,87% of nickel (NiO), 43,70% of Titanium (TiO _2_) and 6,72% of copper (CuO) ( Gravina *et al.*, 2014). These properties explained why the nickel ion released more than the copper ion release within this CuNiTi archwire.

Chlorhexidine used for a long time can generate Reactive Oxygen Species (ROS), the primary agent responsible for endogenous DNA damage ( Septiani and Auerkari, 2020). Nik *et al.* and Omidkhoda *et al.* found that CHX immersion had no significant effect on NiTi archwire surface roughness, corrosion, and frictional resistance ( Danaei *et al.*, 2011; Nik *et al.*, 2013). But some authors found a significant decrease in corrosion resistance due to CHX mouthwashes ( Danaei *et al.*, 2011; Deriaty *et al.*, 2018; Habar and Tatengkeng, 2020), and some authors found more significant surface corrosion with SEM analysis ( Mane *et al.*, 2012; Doddamani, Ghosh and Tan, 2018; Chitra, Prashantha and Rao, 2020). This study also found the surface topography showed that the CHX group had rougher and more pitted surfaces compared to the control group or baseline topography.

In six weeks of immersion, CuNiTi wire in the CHX-NaF group has the highest amount of nickel ions released and in the NaF group, more copper ions were released than the other group. The unloading force difference is also significantly seen in the CHX-NaF and chitosan groups within different immersion durations. As a result, there were changes in CuNiTi mechanical properties after immersion of CHX-NaF, NaF, and chitosan for six weeks.

NaF content in perioKin ^®^ (CHX-NaF) mouthwash can increase the metal ions released from the CuNiTi orthodontic wire. Heravi *et al.* found that the NaF content in mouthwash solution would decrease the corrosion resistance of NiTi and CuNiTi wires as the NaF concentration increased ( Heravi, Hadi Moayed and Mokhber, 2015; Fatene *et al.*, 2019). Sabah and Jarjees also reported that the interaction between fluoride and titanium might cause destruction to the metal coating of the archwires and degrade the mechanical properties ( Sabah, Jarjees and Awni, 2011).

The immersion of chitosan showed significant differences in Nickel and copper ion release, surface topography, and unloading force. Chitosan mouthwash has been used due to its antibacterial effect and lack of side effects. Uraz *et al.* has reported that chitosan 2% mouthwash had no significant difference compared to CHX 0,2% in reducing plaque index and gingivitis ( Uraz *et al.*, 2012).

The highest nickel ion released was found in the chitosan group at week two, but it increased slowly and steadily compared to other groups, and it lasted with the lowest nickel released for the longest immersion time (six-week immersion). The same result with copper ions released. It was found that the chitosan had the lowest amount of ions released from week two until six weeks. Unfortunately, nothing was found about the chitosan particles used as an inhibitory of corrosion for orthodontic archwire, but Putri *et al.* researched orthodontic mini-implant immersed in chitosan mouthwashes for the surface topography found that the mini implant immersed with 1.5% of chitosan mouthwash had a smoother surface compared to chlorhexidine and Sodium Fluoride mouthwash. The CuNiTi archwire surface topography in this study found that the roughness of the chitosan group also had a smoother area compared to CHX and CHX-NaF groups ( Putri *et al.*, 2021).

Furlan *et al.* mentioned that there were differences in the amount of nickel and copper ions released in several types of CuNiTi wire immersed in a neutral solution and an acidic solution, which were greater in the acidic solution ( Furlan *et al.*, 2018). The main limitation of this study was that it did not analyze the pH changes within each immersion solution and each observation time. This study was also done under the static condition *in vitro.* Further research is needed to determine the situation with *in vivo* experiments. The surface structure should also be better analyzed with an advanced equipment, *i.e.,* atomic force microscopy (AFC) so that we could measure the quantity of the surface by its roughness. This will allow us to better understand the physical properties of CuNiTi wire. The inter- or intra-reliability test should appropriately had been suggested for several samples.

## Conclusions

CuNiTi wire had an increase of nickel and reduced copper ion release parallel to the increase of immersion time, but the deflection of the wire did not show any significant differences between mouthwashes. However, after six weeks of immersion, the amount of Nickel and copper ions released was still within the safe limit. The surface roughness of CuNiTi archwire topography showed that the CHX-NaF group had the most extended cracks and deep pits. The unloading force of the CuNiTi archwire deflection remains the same at week two and week four for all mouthwashes.

## Data Availability

OSF: Data of Corrosion of CopperNickelTitanium Archwire in Chlorhexidine, Sodium Fluoride and Chitosan Mouthwashes, https://doi.org/10.17605/OSF.IO/5QB8E ( Sufarnap, 2022). This project contains the following underlying data:
- Copper Ion Release of CuNiTI Tanzo Archwires.pdf - Deflection of CuNiTi Tanzo Archwires.pdf - Nickel Ion Ni Release of CuNiTi Tanzo Archwire.pdf - SEM Surface topography of Tanzo AW (folder containing all raw SEM images) OSF: Data of Corrosion of CopperNickelTitanium Archwire in Chlorhexidine, Sodium Fluoride and Chitosan Mouthwashes, https://doi.org/10.17605/OSF.IO/5QB8E ( Sufarnap, 2022). This project contains the following extended data:
-
Figure 1, 2,4. Graph of Ni-Cu Ion Release and Deflection of Tanzo CuNiTi Archwier.pdf -
Figure 3. Surface Analysis of CuNiTi Tanzo Archwire (SEM analysis).jpeg Data are available under the terms of the Creative Commons Zero “No rights reserved” data waiver (CC0 1.0 Public domain dedication).

## References

[ref1] Aghili H Yassaei S Eslami F : Evaluation of the effect of three mouthwashes on the mechanical properties and surface morphology of several orthodontic wires: An in vitro study. *Dent. Res. J (Isfahan).* 2017;14:252–259. 10.4103/1735-3327.211629 28928779 PMC5553253

[ref2] Alarifi S Ali D Verma A : Cytotoxicity and genotoxicity of copper oxide nanoparticles in human skin keratinocytes cells. *Int. J. Toxicol.* 2013;32:296–307. 10.1177/1091581813487563 23667135

[ref3] Anusavice KJ Shen C Rawls HR : *Phillips’ Science of Dental Materials.* 12th ed. Saunders;2012.

[ref4] Anuwongnukroh N Dechkunakorn S Kanpiputana R : Oral Hygiene Behavior during Fixed Orthodontic Treatment. *Dentistry.* 2017;7. 10.4172/2161-1122.1000457

[ref5] Bhalajhi S : *Orthodontics The Art and Science.* 5th ed. New Delhi: Arya Medi Publishing House;2012.

[ref6] Castro SM Ponces MJ Lopes JD : Orthodontic wires and its corrosion - The specific case of stainless steel and beta-titanium. *J. Dent. Sci.* 2015;10:1–7. 10.1016/j.jds.2014.07.002

[ref7] Chaturvedi T Upadhayay S : No Title. *Indian J. Dent. Res.* 2010;21:275–284. 10.4103/0970-9290.66648 20657101

[ref8] Chen C-Y Chung Y-C : Antibacterial effect of water-soluble chitosan on representative dental pathogens Streptococcus mutans and Lactobacilli brevis, Academia Rd., Nangang Dist. n.d. 10.1590/S1678-77572012000600006 PMC3881855 23329243

[ref9] Chitra P Prashantha GS Rao A : Effect of fluoride agents on surface characteristics of NiTi wires. An ex vivo investigation. *J. Oral Biol. Craniofac. Res.* 2020;10:435–440. 10.1016/j.jobcr.2020.07.006 32817814 PMC7426570

[ref10] Danaei SM Safavi A Roeinpeikar SMM : Ion release from orthodontic brackets in 3 mouthwashes: An in-vitro study. *Am. J. Orthod. Dentofac. Orthop.* 2011;139:730–734. 10.1016/j.ajodo.2011.03.004 21640878

[ref12] Deriaty T Nasution I Yusuf M : Nickel ion release from stainless steel brackets in chlorhexidine and Piper betle Linn mouthwash. *Dent. J (Majalah Kedokteran Gigi).* 2018;51:5. 10.20473/j.djmkg.v51.i1.p5-9

[ref13] Devi I Sufarnap E Finna P : Chitosan’s effects on the acidity, copper ion release, deflection, and surface roughness of copper-nickel-titanium archwire. *Dent. J.* 2022;56:41–47. 10.20473/j.djmkg.v56.i1.p41-47

[ref14] Doddamani GM Ghosh T Tan KFH : Evaluation of degradation in the performance of orthodontic wires after immersing in acidulated phosphate fluoride prophylactic agent: An in vitro study. *Saudi J. Oral Sci.* 2018;5:104–109. 10.4103/sjos.SJOralSci

[ref15] Farrukh MA : *Atomic Absorption Spectroscopy.* First ed. InTech;2011;1–5.

[ref16] Fatene N Mansouri S Elkhalfi B : Assessment of the electrochemical behaviour of Nickel-Titanium-based orthodontic wires: Effect of some natural corrosion inhibitors in comparison with fluoride. *J. Clin. Exp. Dent.* 2019;11:e414–e420. 10.4317/jced.55601 31275513 PMC6599693

[ref17] Fei Liu X Lin Guan Y Zhi Yang D : Antibacterial Action of Chitosan and Carboxymethylated Chitosan. 2000.

[ref18] Furlan TPR Barbosa JA Basting RT : Nickel, Copper, and Chromium Release by CuNi-titanium Orthodontic Archwires is Dependent on the pH Media. *J. Int. Oral Health.* 2018;10:224–228. 10.4103/jioh.jioh

[ref19] Geramy A Hooshmand T Etezadi T : Effect of Sodium Fluoride Mouthwash on the Frictional Resistance of Orthodontic Wires. *J. Dent (Tehran).* 2017;14:254–258. 29296110 PMC5748452

[ref49] Gravina MA Canavarro C Elias CN : Mechanical properties of NiTi and CuNiTi wires used in orthodontic treatment. Part 2: Microscopic surface appraisal and metallurgical characteristics. *Dental Press J. Orthod.* 2014;19:69. 10.1590/2176-9451.19.1.069-076.oar 24713562 PMC4299426

[ref20] Habar EH Tatengkeng F : The difference of corrosion resistance between NiTi archwires and NiTi with additional cooper archwires in artificial saliva. 2020;5:120–123. 10.15562/jdmfs.v5i2.1089

[ref21] Hafez HS Selim EMN Kamel Eid FH : Cytotoxicity, genotoxicity, and metal release in patients with fixed orthodontic appliances: A longitudinal in-vivo study. *Am. J. Orthod. Dentofac. Orthop.* 2011;140:298–308. 10.1016/j.ajodo.2010.05.025 21889074

[ref22] Sabah HH Jarjees HT Awni KM : Effect of Clorhexidine Mouthwash and fluoridated mouthwash on mechanical properties of orthodontic archwires (An in vitro study). *Journal of the 5th Scientific Conference of Dentistry College.* 2011;11:150–159. 10.16194/j.cnki.31-1059/g4.2011.07.016

[ref23] Heravi F Hadi Moayed M Mokhber N : Effect of Fluoride on Nickel-Titanium and Stainless Steel Orthodontic Archwires: An In-Vitro Study. 2015. PMC4436327 26005454

[ref24] IARC Working Group: *International Agency for Research on Cancer. IARC Monograph on the evaluation of carcinogenic risk to human. [WWW Document].* Lyon;1990. (accessed 12.7.22). Reference Source

[ref25] Irmawantini N : *Bahan Ajar Kesehatan Lingkungan Metodologi Penelitian.* Kementerian Kesehatan Republik Indonesia;2017.

[ref50] Khan JA Vu MT Nghiem LD : A preliminary assessment of forward osmosis to extract water from rumen fluid for artificial saliva. *Case Studies in Chemical and Environmental Engineering.* 2021;3. 10.1016/j.cscee.2021.100095

[ref26] Khatri JM Mehta VP : Evaluation of Force Deflection Properties of Various Types of Initial Orthodontic Archwires. *J. Indian Orthod. Soc.* 2014;48:309–312. 10.1177/0974909820140503S

[ref27] Lü X Bao X Huang Y : Mechanisms of cytotoxicity of nickel ions based on gene expression profiles. *Biomaterials.* 2009;30:141–148. 10.1016/j.biomaterials.2008.09.011 18922574

[ref28] Mahalaxmi S : *Materials used in dentistry.* vol.24. Wolters Kluwer;2013; pp.90–91. 10.1016/s0096-6347(38)90036-5

[ref29] Mane PN Pawar R Ganiger C : Effect of fluoride prophylactic agents on the surface topography of NiTi and CuNiTi wires. *J. Contemp. Dent. Pract.* 2012;13:285–288. 10.5005/jp-journals-10024-1138 22917997

[ref30] Marques LS Pazzini CA Pantuzo MCG : Nickel: Humoral and periodontal changes in orthodontic patients. *Dental Press J. Orthod.* 2012;17:15–17. 10.1590/S2176-94512012000200002

[ref31] Metin-Gürsoy G Uzuner FD : *The Relationship between Orthodontic Treatment and Dental Caries.* Intechopen;2014. 10.5772/57353

[ref32] Milheiro A Nozaki K Kleverlaan CJ : In vitro cytotoxicity of metallic ions released from dental alloys. *Odontology.* 2016;104:136–142. 10.1007/s10266-014-0192-z 25549610

[ref33] Mirhashemi AH Jahangiri S Kharrazifard MJ : Release of nickel and chromium ions from orthodontic wires following the use of teeth whitening mouthwashes. *Prog. Orthod.* 2018;19:4–6. 10.1186/s40510-018-0203-7 29399703 PMC5797726

[ref34] Mitchell L : *Introduction to Orthodontics.* Fourth Ed. Oxford University Press;2013.

[ref35] Nik TH Hooshmand T Farazdaghi H : Effect of chlorhexidine-containing prophylactic agent on the surface characterization and frictional resistance between orthodontic brackets and archwires: An in vitro study. *Prog. Orthod.* 2013;14:1–8. 10.1186/2196-1042-14-48 24325758 PMC3895700

[ref36] Parvizi F Rock WP : The load/deflection characteristics of thermally activated orthodontic archwires. *Eur. J. Orthod.* 2003;25:417–421. 10.1093/ejo/25.4.417 12938849

[ref37] Pazzini CA Oliveira G Marques LS : Prevalence of nickel allergy and longitudinal evaluation of periodontal abnormalities in orthodontic allergic patients. *Angle Orthod.* 2009;79:922–927. 10.2319/081408-430.1 19705935

[ref38] Phulari BS : *Orthodontics Principle and Practice.* 2nd ed. Jaypee Brothers Medical Publisher’s;2017.

[ref39] Putri AS Anggani HS Ismaniati NA : Corrosion Resistance of Titanium Alloy Orthodontic Mini-implants Immersed in Chlorhexidine, Fluoride, and Chitosan Mouthwashes: an in-vitro Study. *J. Int. Dent. Med. Res.* 2021;14:996–1002.

[ref40] Roveri N Battistella E Bianchi CL : Surface enamel remineralization: Biomimetic apatite nanocrystals and fluoride ions different effects. *J. Nanomater.* 2009;2009:1–9. 10.1155/2009/746383

[ref41] Sarul M Kawala B Antoszewska J : Comparison of elastic properties of nickel-titanium orthodontic archwires. *Adv. Clin. Exp. Med.* 2013;22:253–260. 23709382

[ref42] Septiani D Auerkari EI : Genotoxicity Effect of Chlorexidine. *Indones. J. Legal Forensic Sci.* 2020;10:46. 10.24843/ijlfs.2020.v10.i01.p06

[ref43] Singh G : *Textbook of Orthodontics.* 2nd ed. New Delhi: Jaypee Brothers Medical Publisher’s;2007.

[ref44] Sivapriya E Sidevi K Periasamy R : Remineralization ability of sodium fluoride on the microhardness of enamel, dentin, and dentinoenamel junction: An in vitro study. *J. Conserv. Dent.* 2017;20:100–104. 10.4103/JCD.JCD_353_16 28855756 PMC5564234

[ref45] Suárez C Vilar T Gil J : In vitro evaluation of surface topographic changes and nickel release of lingual orthodontic archwires. *J. Mater. Sci. Mater. Med.* 2010;21:675–683. 10.1007/s10856-009-3898-7 19826928

[ref46] Sufarnap E Harahap KI Terry T : Effect of sodium fluoride in chlorhexidine mouthwashes on force decay and permanent deformation of orthodontic elastomeric chain. *Padjajaran J. Dent.* 2021;33:74–80. 10.24198/pjd.vol33no1.26370

[ref47] Sufarnap E : Data of Corrosion of Coppernickeltitanium Archwire in Chlorhexidine, Sodium Fluoride and Chitosan Mouthwashes.[Data]. *OSF.* 2022. December 29. 10.17605/OSF.IO/5QB8E

[ref48] Uraz A Boynueǧri D Özcan G : Two percent chitosan mouthwash: A microbiological and clinical comparative study. *J. Dent. Sci.* 2012;7:342–349. 10.1016/j.jds.2012.05.003

