# Supplementary material for: Corrosion of copper nickel titanium archwire in chlorhexidine,  sodium fluoride, and chitosan mouthwashes
Source: F1000Res. 2024 May 30;12:159. Originally published 2023 Feb 10. [Version 3] doi: 10.12688/f1000research.129043.3 (PMC10905017; doi:10.12688/f1000research.129043.3)
Supplement: Peer review history

# Reviewer report: Reviewer response for version 3

**DOI:** 10.5256/f1000research.163146.r284848

**Published:** 2024-08-28

**Authors:** Andrzej Zielinski

**Affiliations:** 1 Gdansk University of Technology, Gdańsk, Poland

After considering all the changes and limitations in the performed research I have no further comments. It is a perfect piece of research, well-written, and it will certainly be of interest. I admire the hard work of the authors in improving the manuscript.

Is the work clearly and accurately presented and does it cite the current literature?

Yes

If applicable, is the statistical analysis and its interpretation appropriate?

Yes

Are all the source data underlying the results available to ensure full reproducibility?

Yes

Is the study design appropriate and is the work technically sound?

Yes

Are the conclusions drawn adequately supported by the results?

Yes

Are sufficient details of methods and analysis provided to allow replication by others?

No

Reviewer Expertise:

Biomaterials engineering

I confirm that I have read this submission and believe that I have an appropriate level of expertise to confirm that it is of an acceptable scientific standard.

# Reviewer report: Reviewer response for version 3

**DOI:** 10.5256/f1000research.163146.r284847

**Published:** 2024-06-04

**Authors:** Asma Ashari

**Affiliations:** 1 Department of Family Oral Health, Faculty of Dentistry, Universiti Kebangsaan Malaysia, Kuala Lumpur, Malaysia

Thank you and no further comment.

Is the work clearly and accurately presented and does it cite the current literature?

Yes

If applicable, is the statistical analysis and its interpretation appropriate?

Partly

Are all the source data underlying the results available to ensure full reproducibility?

Partly

Is the study design appropriate and is the work technically sound?

Partly

Are the conclusions drawn adequately supported by the results?

Partly

Are sufficient details of methods and analysis provided to allow replication by others?

Partly

Reviewer Expertise:

Orthodontics, clear aligners

I confirm that I have read this submission and believe that I have an appropriate level of expertise to confirm that it is of an acceptable scientific standard.

# Reviewer report: Reviewer response for version 2

**DOI:** 10.5256/f1000research.145994.r167825

**Published:** 2024-06-04

**Authors:** Asma Ashari

**Affiliations:** 1 Department of Family Oral Health, Faculty of Dentistry, Universiti Kebangsaan Malaysia, Kuala Lumpur, Malaysia

Under the heading  "Mouthwash immersion phase" Please edit the second paragraph: 

 "Archwires were simulated in the mouth environment, all samples were immersed in 10 ml saliva within observation time (2,4, and 6 weeks), and mouthwashes were simulated two times a day for one minute. **One minute is the recommended time as per manufacturer's instructions**. "

 Under the heading "Experimental analysis and measurement phase" can rephrase second paragraph: 

**"One researcher (Initials) with more than 3 years of experience in AAS, UTM and SEM machines did the measurement of all the samples**. The region of Interest (ROI)....."

Is the work clearly and accurately presented and does it cite the current literature?

Yes

If applicable, is the statistical analysis and its interpretation appropriate?

Partly

Are all the source data underlying the results available to ensure full reproducibility?

Partly

Is the study design appropriate and is the work technically sound?

Partly

Are the conclusions drawn adequately supported by the results?

Partly

Are sufficient details of methods and analysis provided to allow replication by others?

Partly

Reviewer Expertise:

Orthodontics, clear aligners

I confirm that I have read this submission and believe that I have an appropriate level of expertise to confirm that it is of an acceptable scientific standard, however I have significant reservations, as outlined above.

# Reviewer report: Reviewer response for version 2

**DOI:** 10.5256/f1000research.145994.r246726

**Published:** 2024-02-21

**Authors:** Andrzej Zielinski

**Affiliations:** 1 Gdansk University of Technology, Gdańsk, Poland

I have three remarks:

 1. All tests have been performed in saliva or solution containing saliva. It is obligatory to repeat the tests to know the chemical composition of this artificial, presumably, saliva.

 2. The results are positive for all users of archeries, but from a scientific point of view, I am not fully satisfied. Please attempt, it might be even speculative and short, why nickel ions are released relatively quickly, and copper ions are not, scarcely dependent on chemical solution composition.

 3, Twice words are unnecessarily started with capital letters: Chitosan (page 3, bottom of the page), and Nickel (page 9, Conclusions)

Is the work clearly and accurately presented and does it cite the current literature?

Yes

If applicable, is the statistical analysis and its interpretation appropriate?

Yes

Are all the source data underlying the results available to ensure full reproducibility?

Yes

Is the study design appropriate and is the work technically sound?

Yes

Are the conclusions drawn adequately supported by the results?

Yes

Are sufficient details of methods and analysis provided to allow replication by others?

No

Reviewer Expertise:

Biomaterials engineering

I confirm that I have read this submission and believe that I have an appropriate level of expertise to confirm that it is of an acceptable scientific standard, however I have significant reservations, as outlined above.

# Response

**Published:** 2024-05-27

**Authors:** Erliera Sufarnap

**Affiliations:** Universitas Sumatera Utara, Indonesia

Dear Professor Andrzej Zielinski,

 Thank you for allowing us to revised the manuscript. We appreciate the time and effort that you gave and provided us with many valuable comments and feedback to make this manuscript become more clear and great. We were trying our best efforts to make revisions regarding your inquiries.

 The following are our comprehensive three comment’s answers after revised:

 1. All tests have been performed in saliva or solution containing saliva. It is obligatory to repeat the tests to know the chemical composition of this artificial, presumably, saliva.

 Answers:

 Thank you Professor Zielinski for the inquiry.

 The study had been performed with artificial solution presumably most closely to the saliva’s compositions. The solution derived with McDougall’s recipe of minerals with exact ingredient and concentrations which consists of NaHCO3, Na2HPO4.12H _2_O, NaCl, KCl, CaCl2 anhydrous, MgCl2 anhydrous, and H _2_O. We’ve found the original McDougall’s journal about this recipe in detail, but from the year 1947 presumably it had been cited in many journals, and the newest jounal found by Khan et al. 2021 which we were attached to the manuscript.

 Most journal had this recipes made by pharmacian with their own laboratory until now without any modification and also in our hospital pharmacy.

 We were terribly sorry we didn't mention this in the manuscript. We’ve already added it to the manuscript at the “Methods” parts, at Group 1. Here, we also attached the table, cited originally from McDougall’s journal 1947.

Reference Link to Attached table

 2. The results are positive for all users of archeries, but from a scientific point of view, I am not fully satisfied. Please attempt, it might be even speculative and short, why nickel ions are released relatively quickly and copper ions are not, scarcely dependent on chemical solution composition

 Answers:

 The addition of copper (Co) causes a more constant force distribution during activation since the copper has it’s heat conductor function within certain temperature which considered in the mouth. It also gives greater flexibility and exhibits  corrosion resistance (Mitchell 2013, Gravina et al, 2014).

 The mechanical properties of CuNiTi archwire consists 46,87% of nickel (NiO), 43,70% of Titanium (TiO _2) _and 6,72% of copper (CuO) (Gravina et al. 2014). These properties could explained why the nickel ion released were greater than the copper ion release due to Ni properties were greater than Co.

 The chemical solution composition and the acidity would also be a predisposition to greater ion released, for example that the solution which contained a fluoride and also the acid conditioin of the solution would gave more tendency to higher ion released  compared to controls which I already described at the last paragraph from discussion part.

 Thank you Proffessor for the inquiries, I explained about this in discussion session to make the results and discussion more clear.

 3. Twice words are unnecessarily started with capital letters: Chitosan (page 3, bottom of the page), and Nickel (page 9, Conclusions)

 Thank you very much, dear Professor. I corrected the typos in my manuscript by de-capitalizing the letter “chitosan” in many words.

# Reviewer report: Reviewer response for version 2

**DOI:** 10.5256/f1000research.145994.r230212

**Published:** 2024-01-11

**Authors:** SHAZA HAMMAD

**Affiliations:** 1 Mansoura University, Mansoura, Dakahlia Governorate, Egypt

The manuscript was conducted to answer an important issue  concerning the effect of CHX, NaF, and chitosan on the corrosion of  CuNiTi wire nickel and copper ions released, surface roughness change, and archwire deflection..

 The authors modified the manuscript according to previous reviewer’s comments.

 No need for this sentence" All the laboratory assistants at each laboratory have a Master’s degree with above three to seven years of work experience", instead, all is needed is to mention the inter and intra- reliability tests.

Is the work clearly and accurately presented and does it cite the current literature?

Yes

If applicable, is the statistical analysis and its interpretation appropriate?

Yes

Are all the source data underlying the results available to ensure full reproducibility?

No source data required

Is the study design appropriate and is the work technically sound?

Yes

Are the conclusions drawn adequately supported by the results?

Yes

Are sufficient details of methods and analysis provided to allow replication by others?

Yes

Reviewer Expertise:

Orthodontic techniques, materials and appliances.

I confirm that I have read this submission and believe that I have an appropriate level of expertise to confirm that it is of an acceptable scientific standard.

# Response

**Published:** 2024-02-26

**Authors:** Erliera Sufarnap

**Affiliations:** Universitas Sumatera Utara, Indonesia

Dear Professor Hammad

 Thank you for approving our manuscript in F1000Research. We sincerely appreciate you, as the reviewer, who provided your time and effort in giving some feedback to the manuscript. We are very honored and grateful for the insightful comments and valuable improvements to our paper that you gave us. We have already revised and incorporated your inquiries, which we revised in the chapter “Experiment analysis and measurement phase” chapter in the last sentence and the discussion section. These are our revisions. Thank you very much, Prof Hammad.

 Our best regards from Indonesia.

# Reviewer report: Reviewer response for version 1

**DOI:** 10.5256/f1000research.141698.r164307

**Published:** 2023-03-10

**Authors:** Asma Ashari

**Affiliations:** 1 Department of Family Oral Health, Faculty of Dentistry, Universiti Kebangsaan Malaysia, Kuala Lumpur, Malaysia

An interesting study, although it would benefit from a sample size calculation (if no sample size, can mention it as a pilot study), it would add to the strength of the results, since the sample is already quite large.

 Who did the measurements and who scanned the samples? Are they qualified? Was it a single researcher or multiple? Are they calibrated? Any Intra Class Correlation Coefficient to asses intra and inter-reliability.

 "All samples were immersed in 10ml salive within observation time" what does observation time mean? Please elaborate.

 The mouthwashes were immersed for one minute twice a day, although in practice is it not usually advice to do it for 30 seconds? And usually patients do it for less than that. Therefore the effects of this study are maybe twice as much as what you would expect clinically.

 Mouthwashes are swished around the mouth, not immersed, again this may affect the clinical application.

 Shouldn't the wires be washed with saliva instead of with distilled water? since mouthwashes should not be rinsed after gargling.

Is the work clearly and accurately presented and does it cite the current literature?

Yes

If applicable, is the statistical analysis and its interpretation appropriate?

Partly

Are all the source data underlying the results available to ensure full reproducibility?

Partly

Is the study design appropriate and is the work technically sound?

Partly

Are the conclusions drawn adequately supported by the results?

Partly

Are sufficient details of methods and analysis provided to allow replication by others?

Partly

Reviewer Expertise:

Orthodontics, clear aligners

I confirm that I have read this submission and believe that I have an appropriate level of expertise to confirm that it is of an acceptable scientific standard, however I have significant reservations, as outlined above.

# Response

**Published:** 2023-03-22

**Authors:** Erliera Sufarnap

**Affiliations:** Universitas Sumatera Utara, Indonesia

Thank you very much for your kind attention to the wonderful insightful comments on our manuscript. This manuscript became very complete based on your pieces of advice in many chapters. We have revised the manuscript according to some inquiries you have suggested.

 Several additional sentences needed to be added based on the reviewer’s suggestions, which we already provided to consolidate the new sample size formulation.

 The following is our comprehensive comments on the review after revision:

 1. An interesting study, although it would benefit from a sample size calculation (if no sample size, can mention it as a pilot study), it would add to the strength of the results, since the sample is already quite large. The sample size was conducted by using the Federer Technique in our references by Irmawartini as she stated in her book. We added the formulation to the manuscript.

 2. Who did the measurements and who scanned the samples? Are they qualified? Was it a single researcher or multiple? Are they calibrated? Any Intra Class Correlation Coefficient to asses intra and inter-reliability.

 a. The measurement and the scanning process were done by one qualified laboratory assistant (Master's degree) in each lab. Their work experience is above 3 years for AAS and UTM machines and 7 years for SEM machines.

 b. Calibration

 - The SEM machine calibrates twice in a year interval.

 - The AAS machine calibrates once a year interval.

 - The UTM machines were calibrated before each study starts for each day of measurement. The laboratory has 1 to 3 times studies each year.

 c. - The SEM images were taken 3 times for each sample. The SEM images couldn’t be measured objectively. The Region of interest (ROI) of the images captured within the roughest area.

     - The AAS analysis had been taken one time with the Relative Percent Difference (%RPD) below 5%

     - The UTM machine results also came from one-time measurements from the machine. Calibration had been taken 5 times to get the optimal normal curve. The sample which had improper results were recalculated. Due to the 3 time images captured from SEM analysis, the %RPD below 5% for Ions released measurements and the 5 times calibrator had been taken to get the normal curve from the UTM machine measurement, all laboratory assistants were convinced that there was no need any intra or the inter reliability analysis for those inquiries.

 Thank you, Doctor, but we added the above explanation in the manuscript for each part of the analysis in the M&M section.

 3. "All samples were immersed in 10ml saliva within observation time" what does observation time mean? Please elaborate.

 Observation time means the time as each subgroup time. All samples immersed corresponds to subgroup 2, 4, and 6 weeks. Samples were immersed in 10ml saliva within each observation time, acting as a simulation of the daily patient had the archwire within the mouth environment.

 This was our novel method to have a proper simulation of the mouth condition. The archwire did not immerse with only the mouthwash itself which commonly had the high detectable results.

 This method also prevented bad results coming with undetectable measurements if only a short immersed time of each mouthwash at each observation time,  2, 4, and 6 weeks coincidence to 28, 56, and 84 minutes of immersion with the simulation of mouthwash use in 1 minute twice a day.

 4.  The mouthwashes were immersed for one minute twice a day, although in practice is it not usually advised to do it for 30 seconds? And usually, patients do it for less than that. Therefore the effects of this study are maybe twice as much as what you would expect clinically.

 Based on our mouthwash prescription, it is instructed to do the mouthwash twice a day for 1 minute. Therefore, we conduct the immersion time based on it. The condition in which the patient does it for less than the prescribed immersion time is uncontrollable. As a result, we decided on the immersion time as the mouthwash company prescribed.

 5.  Mouthwashes are swished around the mouth, not immersed, again this may affect the clinical application.

 Yes, indeed. Mouthwashes are swished around and mixed with saliva in the patient’s mouth. However, we are using the same amount of mouthwash as it is usually used which is 10mL and mixed with saliva. In addition, the use of an agitator before immersion in the mouthwashes makes the solution homogenous. The samples were once again agitated before the archwire was removed for further analysis. By doing these, hopefully, won’t affect the clinical application.

 6. Shouldn't the wires be washed with saliva instead of with distilled water? since mouthwashes should not be rinsed after gargling.

 While the observation time was ended, the wires have been removed and washed with distilled water to continue for another analysis (surface roughness topography with SEM machine), so that all biological or any material which could contaminate the surface image should be eliminated.                                                                                                                     

# Response

**Published:** 2024-03-09

**Authors:** Asma Ashari

**Affiliations:** Faculty of Dentistry, Universiti Kebangsaan Malaysia, Kuala Lumpur, Kuala Lumpur, Malaysia

I now approve this report
